# School‐Based Access to and Delivery of Oral Health Care

**DOI:** 10.1111/josh.70241

**Published:** 2026-09-29

**Authors:** Mary E. Northridge, Lynn Gargano, Margaret K. Mason

**Affiliations:** ^1^ Department of Dental Medicine Family Health Centers at NYU Langone, NYU Langone Health Brooklyn New York USA

**Keywords:** dental care for children, education, health services accessibility, oral health, quality of life, schools

## Abstract

**Background:**

Given the importance of oral health to overall health, inclusive evidence‐based interventions to improve health care access, quality of life, and learning and development through school‐based delivery of oral health care are needed for all children, including those with special needs.

**Contributions to Practice:**

Integrated delivery of school‐ and community‐based health care holds the potential to improve the health and lives of children and adolescents. Team‐based approaches are effective in ensuring continuity of care on‐site in schools.

**Implications for School Health Policy, Practice, and Equity:**

Greater attention to ensuring socioeconomically disadvantaged students gain access to school‐based delivery of dental services and adolescent priorities (periodontal disease, dental erosion, and misalignment of the upper and lower teeth) may enhance oral health care for these underserved groups. Long‐term tracking of oral health outcomes (caries‐free rate, oral health‐related quality of life) and educational success (attendance/graduation rates, grade point averages) would bolster the case for school‐based delivery of integrated health care.

**Conclusions:**

School‐based oral health programs provide comprehensive on‐site care, including topical fluoride application and sealant placement to prevent dental caries. Involving parents and teachers in program activities emphasizes the importance of oral health to overall health both at home and in school.

At the turn of this century, the first‐ever Surgeon General's Report titled, *Oral Health in America* alerted the public to the full meaning of oral health and its importance to overall health and well‐being, and to the needless pain and suffering endured by population groups who remain unable to share in the success of evidence‐based oral disease prevention and treatment strategies [1]. More than two decades later, the follow‐up report was published titled, *Oral Health in America: Advances and Challenges*, with separate sections devoted to children and adolescents [2]. Among the sobering findings for children were that nearly half were unable to access regular dental care due to economic, social, and geographic barriers, and children with special needs who represent one in five children experienced no improvement in dental care access since the first Surgeon General's Report was released [1, 2]. For adolescents, nearly half‐experienced dental caries (tooth decay), and again, no meaningful improvement was discernable for this age group in over 20 years [2]. Nonetheless, it is notable that dentistry has belatedly turned its attention to this critical life stage, even as most dental providers lack the evidence‐based communication skills (shared decision‐making, motivational interviewing) to effectively engage with adolescents and respond to their self‐identified oral health priorities [3].

In reflecting upon opportunities to maximize the impact of school‐based interventions, the motivational interviewing phrase, “we are all people first” rings true. Nearly a decade ago, one of us (M.E.N.) was an invited speaker at the 2015 Fall Conference of the Oregon Oral Health Coalition in Portland, OR the day after a mass shooting at a local community college. In his introduction, the state dental director, who was eloquent in his grief, asked for her personal “oral health story.” In a heartfelt desire to provide a measure of comfort to him and the other participants, she allowed herself to be vulnerable in public. During the break, she was surrounded by caring attendees asking her questions about her personal oral health experiences rather than the scientific remarks she spent considerable effort preparing and flying across the country to present.

In the same spirit of honesty and humility in front of this school health audience, a brief history of the descriptive epidemiology of dental caries is provided in Table 1 to better ensure that all readers are aware of the central importance of nutrition in promoting oral health.

**TABLE 1:** A brief history of the descriptive epidemiology of dental caries.

Time period	Descriptive epidemiology of dental caries
Earlier human time periods up to 1600 ad	Root caries predominates; occlusal (biting or chewing surfaces) caries are relatively rare since attrition (tooth loss) outpaced occlusal caries
Later centuries of the Early Modern Era (1600 ad to 1800 ad) up to the beginning of the Modern Era (the end of the 19th century)	Introduction of more refined foods and sugar in wealthier countries leads to new caries pattern that is well‐established by the end of the 1800s: caries begin in pits and fissures (natural grooves and hollows on chewing surfaces), and later appear on proximal surfaces (those adjacent to or touching neighboring teeth)
Second Industrial Revolution (late 19th to early 20th century) up to the end of WWII (1945)	Caries experience (both incidence meaning new caries and prevalence meaning existing caries) is higher in wealthier countries than in poorer countries, likely related to diets that are high in refined carbohydrates (processed grains and sugars) in the former
End of WWII (1945) to the present day	Caries is increasingly a disease of poverty due to effective preventive measures (fluoride in its many forms, improved nutrition, regular dental care) available to people in wealthier countries, states, and communities

Before the introduction of sugar in the human diet, teeth were mostly caries‐free.

The three main categories of oral diseases are dental caries, periodontal (gum) disease, and oral and oropharyngeal (throat) cancers [1, 2, 4]. All three of these oral diseases are common chronic conditions that affect a considerable proportion of both child and adult populations worldwide [5]. In terms of the population burden for school‐aged children and adolescents, however, dental caries far surpasses other oral diseases [2, 4, 5]. A growing body of research has found that dental caries can adversely affect children in critical ways, from pain and delayed development to poor self‐image and lack of socialization [2]. To create positive childhood experiences and meaningfully support children as they learn and grow, parents and teachers can serve as role models in preventing and controlling dental caries by demonstrating and supervising effective oral hygiene and dietary practices [2]. See Table 2 for terms and their definitions regarding teeth and dental care in children and adolescents intended to serve as a useful glossary for readers who are unfamiliar with dentistry.

**TABLE 2:** Terms and their definitions regarding teeth and dental care in children and adolescents.

Terms	Definitions
Caries care/management/control	Actions taken to interfere with mineral/tooth loss at all stages of the caries disease process, including non‐operative interventions/treatments, for example, silver diamine fluoride (SDF) application, and operative interventions/treatments, for example, restorations such as dental fillings
Caries‐free	No detectable signs of dental caries
Cavity‐free	No detected cavities in dentin
Cementum	The calcified or mineralized tissue layer covering the root of the tooth which sits inside the alveolar (gum) socket
Demineralization	Loss of tooth mineral due to acids
Dental biofilm	A consortium of microorganisms that stick to a tooth surface, clinically known as plaque
Dental caries	A biofilm‐mediated, diet modulated, multifactorial, non‐communicable, dynamic disease resulting in net mineral loss of dental hard tissues
Dental cavity	A tooth with caries that has progressed far enough to produce a collapse in the integrity of the outer enamel, exposing the inner dentin
Dental enamel	The extremely hard outer covering of the tooth that protects the innermost layers (dentin and pulp) from damage
Dental pulp	The innermost part of the tooth, consisting of connective tissue, blood vessels and nerves
Dental erosion	Progressive loss of dental hard tissue (enamel) by a chemical process that does not involve bacteria and is caused by acids from food/drinks/heartburn or dry mouth/medications
Dental fluorosis	A condition that results from excessive fluoride ingestion during tooth development, resulting in tooth discoloration ranging from white streaks to brown stains and cracks or pits in the tooth enamel
Dental sealants	Thin plastic coatings that protect the tooth and are placed on the occlusal (chewing) surfaces of posterior (back) teeth to prevent caries initiation and stop the progression of non‐cavitated (early) lesions to a point where damage to dental enamel is irreversible
Dentin	The hard, bone‐like portion of the tooth surrounding the pulp, covered by enamel on the crown and cementum on the root
Developmental defects of enamel	Disturbances during the secretion phase result in enamel hypoplasia (less enamel); insults during the mineralization/maturation phases manifest as enamel hypomineralization
Early childhood caries	Early onset of caries in young children with often fast progression which can finally result in complete destruction of the primary dentition (baby teeth)
Minimally invasive care	Dentistry focused on preventing and healing tooth decay without removing any tooth structures
Remineralization	Net gain of mineral in previously demineralized tissue
Sound enamel/dentin	Tooth structure without clinically detectable alterations of the natural translucency, color or texture
Systemic fluoride	Dietary intake of natural substances, including water and food, through inadvertent ingestion of fluoride from dental products such as fluoride toothpaste, and other sources in which fluoride is purposefully added at the community level as a public health benefit
Topical fluoride	All methods of fluoride applied locally to teeth for both self‐care (toothpaste, rinses, gels) and professional use (gels, varnishes, foams, solutions)

The focus on dental caries in this paper is not intended to undermine the value and complexity of identifying and treating potentially devastating oral health conditions such as molar hypomineralization and cleft palate as early as possible to improve the oral health‐related quality of life (OHRQoL) of affected children and adolescents [6, 7, 8].

## 1 Importance of Oral Health to Student Learning and Development

According to the American Academy of Pediatrics (AAP), early childhood caries (ECC) is the most common chronic childhood disease in this country, and if left untreated, interferes with the ability to eat, sleep, speak, play, and learn due to pain and infection [9]. This statement directly connects oral health to the seminal work of Charles Basch and colleagues who argued that healthier students are better learners [10, 11, 12]. It ought to be noted, however, that their 2011 special issue did not include a manuscript related to oral health, which is why the current article is needed.

In terms of an oral health perspective on nutritious meals and snacks for children and adolescents, the American Academy of Pediatric Dentistry (AAPD) joined with other leading health organizations in endorsing plain drinking water and plain pasteurized milk as the best drink options for youths aged 5–18 years [13, 14]. Nutritional advice from the AAPD specific to healthy hydration and oral health is available in Appendix 1 [14].

In making the connections between oral health and student learning and development, obstructive sleep apnea (OSA) provides a vivid example. Briefly, OSA is a sleep disorder characterized by repeated episodes of complete or partial blockage of the upper airway during sleep, resulting in apnea (airflow cessation) or hypopnea (airflow reduction), leading to snoring and daytime sleepiness [15]. Children with OSA have been found to suffer from more dental caries and periodontal disease and poorer OHRQoL than children without reported sleep difficulties, underscoring the value of interprofessional, e.g., medical and dental, collaboration to improve health and education outcomes for children and adolescents with OSA [15, 16].

### 1.1 Advantages of School‐Based Delivery of Oral Health Care

The foremost advantage of school‐based delivery of oral health care is improved health care access for all students attending school, especially when services are available on site during the school day [2, 9, 17]. Such services range from comprehensive oral health care, including prevention (topical fluoride application, sealant placement) and treatment services for dental caries (see also Table 2), to oral health education and promotion of healthy meals and snacks [9, 18]. While focused primarily on oral health, such programs also improve overall health and academic success for enrolled students, and are thus aligned with the Whole School, Whole Community, Whole Child framework of the Centers for Disease Control and Prevention (CDC) [19, 20].

### 1.2 Integration of Medical, Dental, Behavioral Health, and Social Services on Site in Schools

The Community Preventive Services Task Force recommends the implementation and maintenance of school‐based health centers (SBHCs) in impoverished communities to address the social determinants of health, colloquially known as the conditions in which people are born, grow, work, live, and age [21, 22, 23]. In New York City, SBHCs have been operating for over 35 years and are located in more than 317 schools, serving as on‐site health homes for enrolled public‐school children and adolescents [24]. This is the context for the on‐site program that is the focus of this report. To extrapolate the need for school‐based delivery of oral health care to the rest of the United States, oral health surveillance findings are reviewed next.

## 2 Dental Decay in School‐Aged Children and Adolescents in the United States

### 2.1 High Population Burden of Dental Decay in School‐Aged Children and Adolescents

The most recent oral health surveillance report published by the CDC covers the period immediately preceding the coronavirus disease (COVID‐19) pandemic [25]. It provides national estimates for selected oral health measures from the National Health and Nutrition Examination Survey (NHANES), using the NHANES 2017 through March 2020 pre‐pandemic data files [25]. For school‐aged children, dental decay is all too common: nearly half of children aged 6–9 years have experienced cavities in their primary or permanent teeth [26]. Regarding untreated dental decay, nearly 17% of children aged 6–9 years and 10% of adolescents aged 12–19 years had untreated decay during the pre‐pandemic time period January 2017 through March 2020 [26].

The most recent data for the Healthy People 2030 oral health objective OH‐01: *Reduce the proportion of children and adolescents with lifetime tooth decay experience in their primary or permanent teeth*, showed little or no detectable change from the baseline of 48.4% in 2013–2016, to 48.8% in 2017–2020, and remained short of the target of 42.9% [27]. The most recent data for the Healthy People 2030 oral health objective OH‐02: *Reduce the proportion of children and adolescents with active and currently untreated tooth decay in their primary or permanent teeth*, did show improvement from the baseline of 13.4% in 2013–2016, to 12.4% in 2017–2020, but still fell short of the target of 10.2% [27].

Regrettably, but not unexpectedly, evidence from local school‐based oral health programs across states documented considerable unmet need following the suspension of dental services during the COVID‐19 pandemic. The most serious oral health concerns included abscesses and involvement of the pulp (the innermost layer of teeth containing nerves) due to untreated dental caries [28], accompanied by precipitous declines in dental screenings and dental services [29]. The full toll of the COVID‐19 pandemic on the health and well‐being of school‐aged children may never be known, but undoubtably includes substantial pain and suffering for children and adolescents with dental caries [28, 29].

### 2.2 Differential Impact of Dental Decay by Race/Ethnicity and Poverty Status

While differences by race/ethnicity were found for the prevalence of untreated tooth decay in *primary teeth* among children aged 6–8 years (20.9% for Black, non‐Hispanic children and 19.3% for Mexican American children compared to 15.4% for White, non‐Hispanic children), they were not statistically significant [25]. On the other hand, differences by poverty status were pronounced and statistically significant (24.5% for untreated decay in the high and middle poverty status groups compared to 11.6% in the low poverty status group) [25]. The same pattern was found for the prevalence of untreated tooth decay in *primary or permanent teeth* among children aged 6–9 years. For race/ethnicity, the prevalence was 19.0% for Black, non‐Hispanic children and 21.0% for Mexican American children compared to 14.1% for White, non‐Hispanic children, where the differences were not statistically significant [25]. For poverty status, the prevalence was 24.7% in the high and middle poverty status groups compared to 10.0% in the low poverty status group, where the difference was pronounced and statistically significant [25].

Among adolescents aged 12–19 years, untreated tooth decay in *permanent teeth* was statistically significantly higher in older adolescents aged 16–19 years (12.1%) than in younger adolescents aged 12–15 years (8.6%), White, non‐Hispanic (11.7%) and Black, non‐Hispanic (12.9%) compared to Mexican American (6.9%) adolescents, and high poverty status (14.1%) compared to middle poverty status (12.4%) and low poverty status (8.2%) adolescents [25]. Of concern, the most recent data for the Healthy People 2030 oral health objective OH‐09: *Increase the proportion of low‐income youth who have a preventive dental visit*, showed a worsening trend, from the baseline of 75.8% in 2013–2016 to only 68.7% in 2017–2020, far short of the target of 79.9% [27].

### 2.3 Associations of Oral Health and Dental Care With Educational Outcomes

A recent report found that students who needed health care and did not receive it were significantly more likely to miss school due to illness or injury, and the most commonly missed types of health care were dental, medical, and mental health services [30]. Rigorous reviews have either been unable to draw any clear conclusions regarding efforts to promote oral health and educational attainment in school [31] or suggested that while poor oral health may have negative effects on student absenteeism and achievement, study quality was highly variable [32].

A randomized controlled trial of school‐based caries prevention conducted before, during, and after the years of school closures due to the COVID‐19 pandemic found that in years in which treatment was provided, fewer school absences were recorded than in nontreatment years [33]. Nonetheless, the poor reliability of the attendance records resulting from the closing of school facilities in response to the COVID‐19 pandemic may have influenced the results [33].

### 2.4 Effectiveness of School‐Based Interventions to Improve Oral Health

Systemic and topical fluoride administration through community‐ and school‐based programs have been vital in efforts to prevent dental caries in children and adolescents both domestically and globally since the 1940s (Tables 2 and 3) [34, 35]. With greater understanding of the multifactorial, dynamic caries disease process and the ubiquity of fluoride in certain communities and population groups, the best way to deliver this highly effective preventive mainstay and avoid fluorosis (a chronic condition caused by excessive intake of fluorine compounds, ranging from faint white spots to more severe brown stains or pitting in rare cases) deserves the careful consideration of everyone devoted to the health and well‐being of children and adolescents.

**TABLE 3:** Key measures for the Dental School Health Program operated by Family Health Centers at NYU Langone, New York City, updated for school year 2025–2026.

**Demographic Information of Students Enrolled in the Dental School Health Program**
*These fields are available and largely populated in the integrated electronic health record (Epic with Wisdom) in use across all services (dental, medical, behavioral health, and social) and programs of the health center network*
Sex
Age
Race
Hispanic ethnicity
Language
**Program Information Collected and Tracked by Partner School Each School Year Over Time**
Grades served
Inclusion of District 75 programs for students with significant challenges (e.g., autism, cognitive delays, sensory impairments, and emotional or multiple disabilities)
Enrollment numbers, percentages and rates
Utilization numbers, percentages and rates
Co‐location of Family Health Centers at NYU Langone medical and/or behavioral health services
**Baseline Dental Service Measures Collected by Partner School**
Number (percent) of students with untreated caries
Number (percent) of students with non‐cavitated teeth
Number (percent) of students without dental sealants
**Annual Dental Service Measures Collected and Tracked by Partner School Each School Year Over Time**
Number (percent) of diagnostic examinations
Number (percent) of dental prophylaxes (cleanings) to remove plaque (biofilm) and tartar (hardened plaque)
Number (percent) of fluoride varnish applications
Number (percent) of dental sealant placements
Number (percent) of tooth extractions
Number (percent) of tooth restorative services
**Key Dental Service Outcomes Collected and Tracked by Partner School Each School Year Over Time**
Number (percent) of completed treatment plans: Phase 1 (urgent/diagnostic); Phase 1 + Phase 2 (disease control)
Number (percent) of referrals for dental treatment + number (percent) of completed referrals
Number (percent) of referrals for urgent dental treatment + number (percent) of completed urgent referrals
Number (percent) of 3‐year dental sealant replacements
Number (percent) of care transitions from the Dental School Health Program to a community‐based dental clinic
**Key Oral Health Outcomes Collected and Tracked at Intake or Each School Year Over Time**
Caries‐free rate by partner school each school year
Oral health‐related quality of life (OHRQoL) at intake as per the Child Oral Health Impact Profile (COHIP)
OHRQoL annual Global Change Assessment for COHIP domains by student each school year
**Academic/Educational Outcomes Collected and Tracked by Partner School Each School Year Over Time**
Attendance rates
Grade point averages
Graduation rates

School‐based interventions to improve oral health most often target communities and schools with a high burden of disease and a shortage of health professionals [36]. Dental sealant programs are evidence‐based yet underutilized; community partnerships and the training of dental team members in school‐based settings have been found to decrease barriers to care, thereby improving the oral health of public‐school students [37]. The most recent data for the Healthy People 2030 oral health objective OH‐10: *Increase the proportion of children and adolescents who have received dental sealants on 1 or more of their primary or permanent molar teeth*, show that it is getting worse, from the baseline of 37.0% in 2013–2016 to only 25.4% in 2017–2020, even farther from the target of 42.5% [27]. One hopeful explanation for this trend is the increased use of glass ionomer sealants over this time period, resulting in decreased examination sensitivity [38].

Silver diamine fluoride (SDF) is an evidence‐based, minimally invasive intervention to prevent and control caries [39, 40] and has been successfully integrated into school‐based oral health programs [41]. In one report, the quality of life in children receiving SDF was non‐inferior to those receiving sealants and atraumatic restorative therapy at least 6 months post‐treatment and change in OHRQoL did not depend on the severity of baseline dental decay [42].

In summary, dental caries is a disease of poverty. Children living in families with higher levels of resilience and connection were found to have a significantly lower odds of caregiver‐reported dental caries [43]. School‐based oral health programs can relieve pain and suffering and are a necessary but insufficient component of the multilevel interventions that are essential in realizing a caries‐free future for all children and adolescents. A continually evolving program to meet the oral health care needs of New York City public school children is described next.

## 3 Dental School Health Program at Family Health Centers at NYU Langone

The Dental School Health Program (hereafter, the Program) operated by Family Health Centers at NYU Langone currently provides dental homes for largely socioeconomically disadvantaged children at 49 school‐based sites for 62 schools in all five boroughs of New York City [17]. By delivering comprehensive preventive and treatment dental services regardless of the ability to pay to all enrolled students, the Program aims to reduce pain and suffering from oral diseases, improve educational outcomes through fewer missed school days and increased confidence of participants to engage socially, and establish continuity of care throughout the school tenures of students, thus leading to lifetimes of improved oral and overall health for enrolled children. Moreover, the Program addresses the need for greater diversity and community representation among dental providers through the implementation of a workforce development program for dental assistant trainees (BOX 1). In addition, by establishing trusted connections to patients and their families, the Program involves community members in the delivery of oral health care in schools, oral health education in communities, and underrepresented populations in the dental profession. As part of its broad‐based mission, the Program addresses the oral health literacy challenges underscored in the latest Surgeon General's Report [2] through its oral health education, nutrition, and careers in dentistry workshops for middle school students. The overarching goals of these workshops are to inspire early interest in dental careers, reduce disparities in oral health awareness among population groups, and emphasize the critical link between oral and overall health, highlighting how nutritional choices affect lifelong health and dietary habits. Finally, the Program is grounded in the ongoing challenge of dentistry to move from a fee‐for‐service to a value‐based health care (VBHC) system that prioritizes improving patient experiences and outcomes while reducing costs to health care organizations [44].

BOX 1 **Dental assistant training program at family health centers at NYU Langone.**

Despite longstanding calls for more productive dental care, with providers working to the full extent of their licenses as an efficient, productive team, dentistry remains largely siloed. Building a career ladder—with formal progression from entry‐level dental assistant (DA) positions to higher levels of education, responsibility, salary, and skill—may be an important strategy for increasing dental workforce diversity and addressing shortages. DAs traditionally perform a variety of activities depending on the state, including taking health histories, imaging, teaching health promotion techniques, performing office management tasks, communicating with patients and suppliers, and assisting dentists with procedures. At Family Health Centers at NYU Langone, a Federally Qualified Health Center network centered in the Sunset Park neighborhood of Brooklyn, NY, DAs also serve as trusted front‐line staff and liaison with parents and other key stakeholders in its extensive Dental School Health Program consisting of 62 public schools, including four District 75 programs that serve students with disabilities, in all five New York City boroughs. These school‐based clinics serve as dental homes providing comprehensive care on site for participating children. In response to the longstanding DA workforce diversity and shortage challenges that were exacerbated by the coronavirus disease (COVID‐19) pandemic, Family Health Centers at NYU Langone instituted a dental workforce development program supported by community board members and school leaders with two tracks that are free of charge to participants: (1) registered DA trainee (RDAT); and (2) DA trainee (DAT). Both tracks include didactic courses (online sessions offered through Monroe Community College) and clinical components (mentorship by participating dentists), with RDATs/DATs assigned to Family Health Centers at NYU Langone school‐ and community‐based dental clinics.

To illustrate its effectiveness, as part of the Healthcare Equities Task Force at Family Health Centers at NYU Langone, a comprehensive, multilevel approach was used to reduce disparities in dental sealant placement between English‐ and non‐English‐speaking pediatric patient populations, especially for Spanish‐ and Chinese‐speaking parents/patients who constitute the largest non‐English‐speaking patient groups at the health center. By far the largest contributor to sealant placement at Family Health Centers at NYU Langone is the Dental School Health Program. Dental sealant completion rates for Spanish‐speaking parents/patients improved from 84% in April 2024 to 86% in March 2025 and dental sealant completion rates for Chinese‐speaking parents/patients improved from 75% in April 2024 to 80% in March 2025. Over the two‐year course of the project, the benchmark for sealant compliance was raised from 78% in 2023 to 80% in 2025, reflecting sustained progress in reducing language disparities in dental sealant placement. Preliminary findings indicate that the incorporation of “smart phrases” in the integrated electronic health record for new patient examinations and recall visits strengthened provider adherence to the “single visit” sealant protocol, decreasing “no‐show” rates for follow‐up pediatric dental appointments.

### 3.1 Ecological Model

The theoretical basis for the Dental School Health Program is summarized in the *Ecological Model to Improve Student Learning through School‐based Delivery of Oral Health Care* based on earlier work by the authors [45] (Figure 1).

**FIGURE 1:**
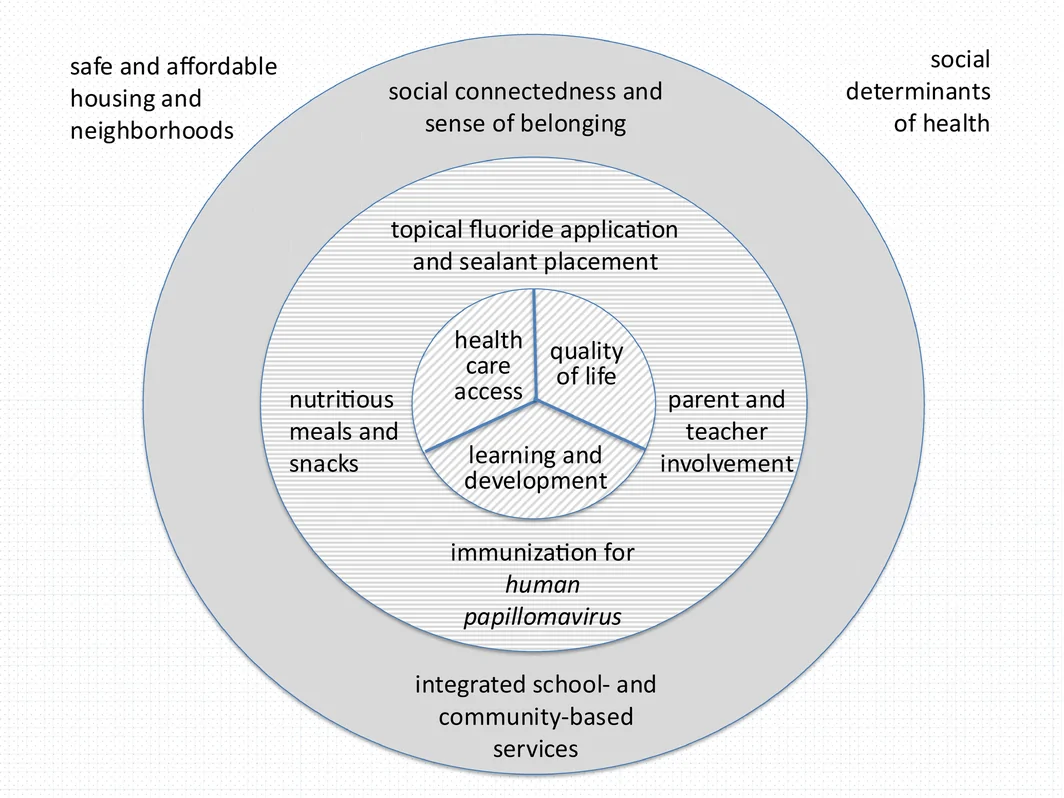
Ecological Model to Improve Student Learning through School‐based Delivery of Oral Health Care. An ecological model of influences on health care access, quality of life, and learning and development at the student level (innermost circle of the figure), brought about by determinants at the Dental School Health Program level (middle concentric circle of the figure), school/community levels (outermost circle of the figure), and local/state/societal levels (square that encompasses all levels of the figure), adapted from previous work by the authors [45] and updated to focus on evidence‐based interventions embraced by the NYU Langone Dental Medicine Postdoctoral Residency programs.

It was updated based upon a literature search to focus on evidence‐based interventions embraced by the NYU Langone Dental Medicine Postdoctoral Residency programs (Appendix 2).

School‐based delivery of oral health care is necessitated by the longstanding and considerable unmet need for quality oral health care in disadvantaged populations served by SBHCs [4, 19, 21, 23]. The problem is exacerbated since oral diseases and craniofacial injuries are at elevated rates throughout the life course in historically marginalized children and adolescents due to determinants at multiple levels [4, 46]. A corollary is that evidence‐based interventions may improve health care access, quality of life, and learning and development at the student level (innermost circle of Figure 1) through strategies at the Program level (middle concentric circle of Figure 1), school/community levels (outermost circle of the figure), and local/state/societal levels (square that encompasses all levels of Figure 1). For example, dental providers trained in motivational interviewing at the Program level are better able to communicate with vaccine‐hesitant parents/guardians and increase the uptake of immunization for *human papillomavirus* (HPV) for both boys and girls [47], thereby effectively preventing oropharyngeal (throat) and oral cancer among both men and women [48]. As another example, at the school/community levels, whole school approaches [19, 20] are among the promising strategies to promote school connectedness for adolescents [49].

### 3.2 Key Measures

The key demographic, service, and outcome measures tracked by partner school over time for the Program are listed by category in Table 3.

This listing is not intended as a standard for other school‐based oral health programs to follow, but rather as a pragmatic approach by the authors and Program staff to track successes and setbacks in meeting identified goals and established benchmarks over time.

The selected demographic fields are those that are available and largely populated in the integrated electronic health record (Epic with Wisdom) currently in use across all services (medical, dental, behavioral health, and social) and programs of NYU Langone Health, of which Family Health Centers at NYU Langone is a constituent service provider. Newer demographic fields may be added to the key measures once missing data are minimized due to ongoing quality improvement initiatives and sufficient elapsed time for meaningful tracking.

The two oral health outcome measures (caries‐free rate by partner school and OHRQoL) merit further explanation. First, the caries‐free rate of the population of enrolled children as a percentage of the entire population of each school served annually is considered to be a sensitive measure of the oral health of a school population [50, 51, 52, 53, 54]. Due to the complex relationships among student, Program, school/community, and local/state/societal determinants of caries and their prevention and treatment (Figure 1), tracking is conducted within schools over time rather than between schools. Second, the Child Oral Health Impact Profile (COHIP) was designed to assess children's oral‐facial well‐being (OHRQoL) in school‐aged children as reported by both the child and via proxy report from a caregiver [55]. The COHIP measures how oral health affects daily activities such as speaking, eating, smiling, learning, and emotional/social well‐being. It consists of 35 items forming five conceptually distinct domains: oral health, functional well‐being, social/emotional well‐being, school environment, and self‐image [56]. The Global Assessment of Change consists of five questions specific to each of the five domains of the COHIP (e.g., for oral health, “Overall, has there been a change in the health of your teeth or mouth since your last interview?”) as well as a question regarding total health (“In general, has there been a change in your overall health since your last visit?”) [57]. These outcome measures are proposed to be collected by Program staff with enrolled children and their parents in pilot schools before implementing the workflow changes in the electronic health record used throughout NYU Langone Health at all of its constituent sites, including schools. Based on the reviewed surveillance findings and our own programmatic experiences, a discussion of the implications for school health research and ongoing efforts to provide equitable access to oral health care in New York City and more broadly is provided next.

## 4 Discussion

### 4.1 Implications for School Health Research

Despite decades of scientific advancements in the prevention and treatment of oral diseases, little progress has been made for students served by school‐based oral programs, especially children with disabilities and adolescents. Most of the challenges encountered in providing on‐site oral health care for District 75 students with significant challenges, such as autism, cognitive delays, sensory impairments, and emotional or multiple disabilities [58], are logistical in nature (e.g., scheduling around caregiver availability, ensuring sensory‐friendly environments and accessibility and space for specialized equipment in examination rooms). Implementation science approaches may be helpful in overcoming these setbacks to care provision.

A new model of adolescent oral health care that addresses both emerging health risks (substance use, sexual activity) and the desire for personal agency in this age group has been proposed by the authors [59]. By blending orthodontics, self‐image, and behavior change via meaningful provider—patient relationships, the Dental School Health Program hopes to capitalize on regular maintenance visits to offer evidence‐based strategies (e.g., immunization for HPV, shared decision‐making to decrease consumption of sugary drinks) to activate and inform adolescents during this critical life stage for establishing independent and positive lifelong habits. Including the perspectives of population groups who have been traditionally marginalized may help in the design of oral health care delivery systems that are responsive to their needs [60].

Recommendations for school health research include: (1) engage community stakeholders in intervention development, implementation, and evaluation; (2) train teachers, parents, and community activists to humanistically counter myths regarding the effectiveness of fluoride in preventing and reversing carious lesions and vaccines in preventing oropharyngeal cancer; (3) collect and report longitudinal educational outcome data, which is especially important to policy makers; and (4) strengthen the scientific approaches used to integrate school health services, including implementation science and community‐based participatory research [61].

### 4.2 Barriers to School‐Based Oral Health Care Delivery

The historical and continued separation of the oral health care delivery system from the medical care delivery system in the United States is comprehensive, including for school‐based delivery of services [4]. A mindset that acknowledges that oral health is a critical component of overall health is essential in designing and implementing integrated health care systems. In addition, identifying key oral health outcomes and suitable measures that can be tracked over time and place for children and adolescents is an urgent priority in moving dentistry from fee‐for‐service to a VBHC system and strengthening the evidence base for improving student learning through school‐based oral health care delivery [44, 46].

### 4.3 Limitations

There are at least four major limitations to this Practitioner's Perspective article. First, there is a dearth of data linking school‐based access to and delivery of oral health care to learning outcomes in the extant literature as well as in the Program to date. Moving forward, collaboration with the New York City Department of Education will be indispensable in tracking academic/educational outcomes by school (attendance rates, grade point averages, graduation rates). Second, a comprehensive search of the scientific literature, authoritative government reports, and oral health foundation white papers was not conducted. Third, the results emphasize recent evidence published on school‐based oral health programs and projects in the United States. And fourth, the experiences of the authors in designing, implementing, and evaluating school‐based oral health programs are largely limited to sites operated by dental schools, hospitals, and community health centers in impoverished communities in New York City, and may not be generalizable to other contexts.

## 5 Implications for School Health Policy, Practice, and Equity

### 5.1 Strategies to Maximize the Impact of School‐Based Interventions

Foremost among strategies to maximize the impact of school‐based interventions are identifying trusted partners and messengers to ensure that children are enrolled in evidence‐based programs such as the Dental School Health Program [17]. Potential policy solutions would do well to focus on improving the nutritional quality of children's diets in schools and their surrounding neighborhoods, while also expanding the capacity of Federally Qualified Health Centers such as Family Health Centers at NYU Langone to deliver quality oral health care services on site [17, 45].

In terms of practice‐based strategies, advancing the oral health literacy of parents, teachers, school health nurses, community health workers, and other trusted messengers in impoverished schools to deliver timely, understandable, and actionable information better ensures their own oral health, and further, the oral health of their families and communities [2]. Finally, school‐based access to and delivery of oral health care holds the promise that all students may benefit from the services offered, thereby advancing oral health equity [4].

## 6 Conclusions

### 6.1 Challenges Translating Existing Evidence Into Policy Change

“Think globally, act locally” is a reminder that achievable steps in delivering on‐site oral health care prevention and treatment services in neighborhood schools can translate into seismic shifts in the burden of oral diseases in socially disadvantaged groups, thereby promoting health equity and lifelong improvements in population health and well‐being [62].

## Funding

This work was supported by Health Resources and Services Administration (H80CS07914, D88HP37549), New York State Department of Health (C38355GG), Mother Cabrini Health Foundation (2025‐8584), New York City Department of Health and Mental Hygiene, and New York City Department of Education.

## Conflicts of Interest

The authors declare no conflicts of interest.

## Data Availability

Data sharing is not applicable to this article as no new data were created or analyzed in this study.

## Appendix 1 Nutritional Advice for Healthy Hydration and Oral Health

The American Academy of Pediatric Dentistry (AAPD) provides the following tips for healthy hydration specific to oral health on its webpage titled, *Spring Into Healthy Hydration: The Best Drinks for Kids' Smiles and Overall Health*.
And if they do enjoy a sweet drink occasionally, have them rinse with water or brush their teeth afterward to wash away harmful sugars. Making Healthy Hydration Fun This Spring Hydration doesn't have to be boring! Get kids excited about making healthy drink choices by giving them their own reusable water bottles in fun spring colors or patterns. Try adding slices of fresh fruit to their water for a refreshing twist without added sugar. At snack time, pair a glass of milk with crunchy, fiber‐rich foods like apples or carrots, which naturally help clean teeth.

## Appendix 2 Search Strategy

Following is the search strategy used for identifying peer‐reviewed studies on the topic, School‐based Access to and Delivery of Oral Health Care.

The first author (M.E.N.) conducted preliminary literature searches from July 2025 through September 2025 in two major databases: PubMed and Google Scholar. A combination of terms were used in these literature searches on both platforms, including but not limited to: “school‐based health care,” “school health programs,” “school‐aged children,” “school‐aged adolescents,” “school oral health services,” “school dental services,” AND “educational outcomes,” “academic outcomes,” “dental service outcomes,” “oral health outcomes,” “oral health equity.” Manual review of reference lists from relevant articles also yielded publications for inclusion in this paper. Given the focus of this Practitioner's Perspective article on oral health care for children and adolescents, websites and gray literature from well‐established oral and general health care organizations and government agencies were also included in the research presented in this report. A narrative synthesis approach guided the integration of the literature to identify key aspects of the issue and opportunities for further research and advocacy. Up‐to‐date evidence specific to oral health care and school‐based programs published in the United States was prioritized for inclusion.
